# Risk of Transverse Myelitis Following Dengue Infection: A Systematic Review of the Literature

**DOI:** 10.3390/pharmacy7010003

**Published:** 2018-12-23

**Authors:** Nafisa Badat, Dalia Abdulhussein, Peter Oligbu, Olakunle Ojubolamo, Godwin Oligbu

**Affiliations:** 1 School of Medicine, Imperial College London, London SW7 2AZ, UK; nafisa.badat13@imperial.ac.uk (N.B.); da1813@ic.ac.uk (D.A.); 2 Department of Family Medicine, University of Benin Teaching Hospital (UBTH), Benin City, Nigeria; petolis2008@gmail.com; 3 Department of Medicine, Queen’s Hospital, Romford RM7 0AG, UK; olakunle.ojubolamo1@nhs.net; 4 Paediatric Infectious Diseases Research Group, Institute for Infection and Immunity, St. George’s, University of London, London SW17 0RE, UK

**Keywords:** Dengue Fever, transverse myelitis, risk, systematic review

## Abstract

**Introduction:** Dengue virus (DENV) is one of the most common arbovirus diseases, with a wide spectrum of presentation. Spinal cord involvement in dengue infection (DF) is rare. However, the risk of transverse myelitis (TM) following Dengue has not been systematically assessed. **Methods:** We undertook a systematic review of published English literature from January 1974 to December 2017 to assess risk of TM and outcomes following DF. Data sources included EMBASE, MEDLINE, Cochrane library, ISI web of knowledge, conference proceedings and references within identified articles. **Results:** We identified 242 potential studies, 62 were duplicates. A further 136 were excluded on the basis of title and abstract and 19 studies did not meet the eligibility criteria on full text screening. We included 25 publications involving 2672 cases of DF. A small proportion (10.8%, (289/2672)) had neurological complications, of which 2.3% (61/2672) was TM. For articles reporting epidemiological data, the neurological complication was twice in males compared to female 67.7% (130/192) vs. 32.7% (62/192) and 1.5-fold increase TM for males 59.3% (32/54) vs 40.7% (22/54). The mean age at presentation was 33.1years (range 0.75–61), with onset at 11.7days. The method of diagnosing TM due to DF was mainly IgM seropositivity 92% (n = 23/25) and the commonest treatment modality was steroid 78.3% (n = 18/23). Only half had full recovery 50.8% (n = 31/61). There was no mortality following dengue, however, the crude case fatality rate following TM was 3.3% (n = 2/61). **Conclusion:** This review highlights the risk of TM following dengue. Although neurological complications are rare, especially TM, once set in, it is associated with a significant morbidity.

## 1. Introduction

Dengue is a viral disease transmitted by the Aedes mosquito and is endemic in tropical and subtropical areas, in particular the Americas and Asia. This puts an estimated 4 billion people at risk of acquiring the virus; currently it is estimated 100 million cases of symptomatic dengue occur annually [1]. Lack of treatment and immunisation therapy, as well as inadequate vector control have meant that there are no options in the management of severe disease apart from supportive measures [1,2]. In addition, with population growth and increased intercontinental travel over the past decade, it is more likely, if no other combative measures are utilised, that the number of cases will continue to increase.

The vast majority of cases are asymptomatic [1], and where symptoms do occur, these commonly manifest with a fever, generalised pain, nausea and vomiting [2,3]. Severity of infection has been traditionally assessed by cardiovascular compromise, but most recently is the addition of central nervous system (CNS) involvement as a factor of severity since the number of cases describing dengue neurotropism have come to light [2,3]. This may be because factors contributing to neurological manifestations are themselves of increased severity of disease, for example prolonged shock, hepatic failure and hyponatraemia [4,5,6]. 

Damage to the spinal cord (myelitis) following infection can occur during infection (parainfectious) via direct invasion, or after infection (postinfectious) via a proposed immune-mediated inflammatory process [3,7,8]. Transverse myelitis has been described in a number of case reports where the main manifestations are sensorimotor disturbance of the lower limbs and urinary retention [7,9,10,11,12,13,14,15]. Currently the mechanisms of spinal cord damage in dengue are poorly defined and the exact burden of these neurological manifestations is yet to be fully assessed. Hence, we summarised the literature on the risks of transverse myelitis following dengue infection as well as the proposed mechanisms behind this. 

## 2. Methods

### 2.1. Data Search and Selection

A search was designed to identify case reports and observational studies (case series, cohort study, case–control study) reporting transverse myelitis as a complication of dengue viral infection. It aimed to include all publications that evaluated the current data in use of the risk of transverse myelitis (TM) following dengue virus infection globally. We searched MEDLINE, ISI web of knowledge, conference proceedings and EMBASE from 1 January 1974 to 26 December 2017. Both free text and the use of medical subheadings (MeSH) terms were used as search items. An initial search was conducted in order to scope all appropriate search terms followed by a more extensive search using two similar search criteria. The MeSH terms and free text terms used are included in the Appendix A.

Studies were excluded if they were individual opinion or non-availability of full text, experimental or laboratory studies or not original research. We only included studies published in English language in our review. After the initial screening process, all publications were assessed for eligibility based on their titles followed by abstracts and full text.

### 2.2. Study Selection

Studies were eligible for inclusion if they reported neurological complications following dengue infection which were relevant to our study focus; to review the risk of TM following dengue infection. Articles irrelevant to the study were excluded or if they didn’t mention the risk of TM in relation to dengue infection.

Two reviewers (G.O. and N.B.) independently screened the titles and abstracts of papers identified by the electronic searches, evaluating exclusion and inclusion criteria for all papers. We retrieved full text articles of included publications and each was then independently reviewed for eligibility.

### 2.3. Quality Assessment and Data Extraction

Two independent reviewers (G.O. and N.B.) reviewed the methodological quality of included studies, the comparability of case and controls, and outcomes. The explanatory variables extracted included: country, study design, description of study subjects, underlying comorbidity, clinical presentation, management and the outcome of the patient with TM. The study quality assessment was undertaken using the Reporting Items for Systematic Reviews and Meta-analyses (PRISMA) statement for the conduct and reporting of systematic reviews [16].

### 2.4. Data Analysis

Eligible studies were summarised using descriptive analyses to provide the overview of the information on populations studied, clinical presentations, underlying comorbidity and patient mortality outcomes. We calculated the age and sex distribution of TM generalised from the extracted data. We also calculated the risk of TM following dengue infection in children following dengue infection and compared this with that obtained in the adults for the outcome of interest where data were available. We calculated the crude fatality rate as the total number of mortality following TM divided by total number of reported TM cases over the same period. Eligible studies were then analysed qualitatively using Microsoft Office Excel 2007 and summarised.

## 3. Results

**Figure 1:**
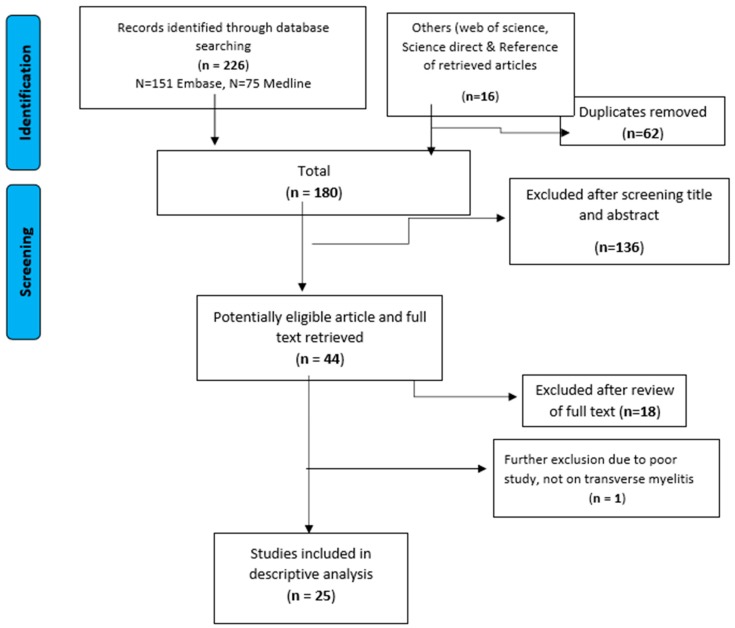
Identification and the selection of eligible studies in the systematic review.

We identified 242 potential studies, of which 62 were duplicates. A further 136 were excluded on the basis of title and abstract and 19 studies did not meet the eligibility criteria on full text screening (Figure 1). The remaining 25 studies were eligible and full text was assessed for inclusion in the final review [7,9,10,12,13,14,15,17,18,19,20,21,22,23,24,25,26,27,28,29,30,31,32,33,34]. Most of the studies were from Asia (76%; 19/25) and the rest from South America (24%; 6/25). The majority of the studies were case reports (64%; 16/25), case series (8%; 2/25), cohort study (16%; 4/25), cross-sectional studies (8%; 2/25) and one prospective study (4%; 1/25). Only five studies reported dengue serotype; three studies had serotype 1 only, serotype 2 only, and serotype 3 only. The other two studies displayed had either dengue serotypes 1–3, or all four serotypes. A summary of the study design, study subjects, data collection method, and treatment is presented in Table 1 and Table 2. Most of the included studies did not report the ethnicity. 

A total 2672 cases of Dengue fever in all ages involving 289 (10.8%; (289/2672)) with neurological complications in 25 studies were included in the final analysis (Table 2).

Overall 2.3% (61/2672) had TM, and children (<18 years old) constituted 13% (8/61) of TM cases reported by six studies. Twenty-two studies reported epidemiological data; the neurological complication was twice in males compared to female 67.7% (130/192) vs. 32.7% (62/192) and 1.5-fold increase TM for males 59.3% (32/54) vs. 40.7% (22/54). The mean age at presentation was 33.1years (range: 0.75–61). Of the 19 papers reporting the onset of DF to the time it was complicated by TM, the average was 11.7days (range: 5–42).

All the studies reported method of diagnosing TM, and apart from the use of radiological investigation by all the studies, the method of diagnosing TM due to DF was mainly IgM seropositivity (92% (n = 23/25)). In addition, 12 papers mentioned additional methods were also used in diagnosing cases of TM; cerebrospinal fluid analysis (CSF) analysis (nine studies), IgG antibodies (two studies), clinical features and nonstructural protein 1 (NS1) antigen assay (two studies) with one other study which used an antibody index ratio of IgM to IgG.

Out of the 25 studies, 92% (n = 23/25) specified their management plans. High dose methylprednisolone was used in 82.6% (n = 19/23) of studies with additional antibiotic cover. Twenty-two per cent (n = 5/23) of studies required in addition intravenous immunoglobulins, of which two had assisted ventilation and one had blood/platelet transfusions. Three studies employed a symptomatic management plan. Only one study treated with antibiotics only and a laminectomy was a modality of management in one of the studies.

The commonest treatment modality was steroids 82.6% (n = 19/23). In terms of recovery after, only half had full recovery 50.8% (n = 31/61) from TM. There was no mortality following dengue infection reported, however, the crude case fatality rate following TM was 3.3% (n = 2/61), involving a 45-year-old male and a 9-month-old male infant. 

## 4. Discussion

A detailed systematic review of the literature identified all reported cases of TM following DF in endemic countries irrespective of the mode of presentation. Overall there were 61 cases in the literature, accounting for 2.3% of DF and the crude case-fatality rate among TM cases was very low at 3.3%. These findings, contrary to previously reported rare occurrence, confirm the prevalence of TM following Dengue. Moreover, Dengue is the most common arboviral disease [35], and occurs in Southeast Asia, East and West Africa, the Caribbean and the Americas [36]. Interestingly, the majority of TM cases were in Asia and a few reported cases in North America. One of the explanations for the low prevalence of TM following DF in West Africa and the Caribbean is that the Asian population appears to be prone to autoimmune injury of the spinal cord and some genetic make-up, including the type of Dengue that causes TM might be different and their contributing factors. Although, neurological complications in dengue fever have been documented with all serotypes, we also observed that it is more common with serotypes 2 and 3 [33].

For example, of the four strains of dengue virus implicated in the disease, DEN3, DEN2 and DEN1 are the prominent serotypes in India. The DEN2 has been reported in more than 75% of the cases in breakouts since 2010 [37,38]. A similar finding was observed in the review with serotype 2 having been the most commonly isolated, however, this was only reported in five of the studies. 

The mechanisms of viral transmission and spinal cord injury induced by dengue virus are unclear. Two mechanisms have been postulated: by direct invasion of the cord and by active replication within the spinal cord [13], which is common during the early phase or postinfectious immune injury [39]. Since only five studies were able to isolate dengue IgG/IgM or antigen in the CSF, it is therefore most likely that both mechanisms have been implicated in the cases in this review. 

One important finding is the 2-fold increase in neurological complication, and a 1.5-fold increase in those that had TM in females compared to males. In addition, the two mortalities were in males. This supports the earlier studies indicating that other factors including biology, environment and experience are contributors to human health [40], but contrary to the reports that most autoimmune diseases are more frequent in females than in males [41].

There is currently no agreed consensus on the management of TM. Our findings showed that almost 80% cases were treated with high dose of methylprednisolone despite insufficient evidence regarding the utility of steroids in treating transverse myelitis [42]. It is therefore advisable that until more robust evidence is available, administration of high dose intravenous (IV) methylprednisolone will be the first treatment of choice in TM to enhance neurological functions. Few cases however required immunoglobulins but this was introduced at a later stage, and to those cases that are presumed to be very sick, thus assessing the efficacy at this stage becomes difficult. This has been considered mainly as second line therapy in patients who have not recovered or are poorly recovering from TM [42].

Due to the supposedly rarity of TM associated DF, there has been controversy as to the actual prevalence of TM following DF. de Sousa AM [9] and colleagues in a retrospective study conducted in the Brazilian Amazon region showed almost half of all DF cases had TM following DF (44%, 26/59). This was adduced to an epidemic of DF at the time of the study compared to the study in a tertiary centre in India where of the 116 patients with DF only 1% had TM. Our review of 2.3% of TM-associated DF may have been underestimated and should therefore be interpreted with caution, since some post infectious TM have been known to present even months after the primary DF infection [23,31]. More importantly, is the significant morbidity associated with TM following DF, as only half had a full recovery from TM before discharge with 19.7% with no reported recovery. This highlights the need for a careful evaluation of patients with DF for TM and other possible neurological complications, and prompt management as high dose steroids has shown to be effective, especially if instituted early in the management of suspected cases of TM following Dengue.

However, our results demonstrate the strengths of combining outcomes of rare events through a detailed systematic review of the literature. The large number of case reports and lack of observational studies was a limitation; consequently, we were unable to conduct any meta-analyses to compare differences in other TM-associated neurological complications or calculate risks associated with clinical outcomes. In addition, as would be expected from case reports, several of the population denominators were not available to identify cases; this could potentially lead to double counting of the same cases. Therefore, it is important that future studies report the number of cases of DF during the time period so that TM rate can be calculated and compared in different population.

## 5. Conclusions

This review highlights the risk of TM following dengue. Although neurological complications are rare, especially TM, once set in, they are associated with significant morbidity. A high index of suspicion is therefore required with careful evaluation and follow-up of patients, as well as, prompt management to enhance recovery.

## Figures and Tables

**Table 1:** Description of the study design and the reported outcomes.

Study	Year of Study	Country	Study Design	Number of Dengue Cases	Diagnosis Method	Outcome
Singh et al. [17]	2013	India	Case Report	1	Dengue IgM seropositivity	1 patient died.
Ghosh et al. [18]	2011	India	Case Report	1	Dengue IgM seropositivity CSF analysis	full recovery
Seet et al. [7]	2006	Singapore	Case Report	1	Antibody index ratio of dengue IgM:IgG	full recovery
Kunishige et al. [13]	2004	Singapore	Case Report	1	Dengue IgM seropositivity (CSF)	partial recovery
Fong CY et al. [19]	2016	Malaysia	Case Report	1	Dengue IgM seropositivity	full recovery
Gupta et al. [20]	2013	India	Case report	1	Dengue IgM seropositivity (ELISA) History NS1 ag assay	full recovery
Wasay et al. [21]	2008	Pakistan	Case Series	6	Dengue IgM seropositivity	4 patients made a full recovery, 2 patients died.
Samanta et al. [22]	2012	India	Case Series	3	Dengue IgM seropositivity (serum/viral/blood)	1 patient made a full recovery, 1 patient made a partial recovery, 1 patient died.
Misra et al. [23]	2015	India	Case Study	116	Dengue IgM seropositivity History, Exam, NS1 antigen assay	78 patients made a full recovery, 27 patients made a partial recovery, 11 patients died.
Sahu et al. [24]	2014	India	Cohort	484	Dengue IgM seropositivity	479 patients made a full recovery, 5 patients died
Soars et al. [25]	2006	Brazil	Cross-sectional study	13	Dengue IgM seropositivity (blood/CSF (ELISA))	12 patients made a full recovery, 1 patient with encephalitis died
Weeratunga et al. [26]	2014	Sri Lanka	Cross-sectional Study	7	Dengue IgM seropositivity (blood/CSF)	6 patients made a full recovery, 1 patient made a partial recovery.
Puccioni-Sohler et al. (Brazil) [12]	2009	Brazil	Retrospective study	27	Dengue IgM seropositivity	Partial recovery
Larik et al. [10]	2012	Singapore	Case Report	1	Dengue IgM seropositivity Dengue RNA	Full recovery
Lim et al. [27]	2012	Singapore	Case Report	1	Dengue IgM seropositivity	Partial recovery
Tomar et al. [28]	2015	India	Case Report	1	Dengue IgM seropositivity	Full recovery
Mo et al. [29]	2016	China	Case Report	1	Dengue IgM/IgG seropositivity (CSF)	Partial recovery
Mota et al. [30]	2017	Brazil	Case Report	1	Dengue IgM seropositivity	Partial recovery
Leão et al. [14]	2000	Brazil	Case Report	1	Dengue IgM seropositivity (CSF)	Full recovery
Miranda de Sousa A et al. [31]	2014	Brazil	Case Report	1	Dengue IgM seropositivity (CSF)	Full recovery
Renganathan et al. [32]	1996	Malaysia	Case Report	1	Dengue IgM seropositivity	Full recovery
Chanthamat et al. [15]	2010	Thailand	Case Report	1	NA	Full recovery
Solomon et al. [33]	2000	Vietnam	Prospective Study	1675	IgM/IgG seropositivity (CSF)	Partial recovery
Sousa et al. [9]	2004	Brazil	Retrospective Study	51	Dengue IgM seropositivity (CSF)	49 patients made a full recovery, 2 patients had partial recovery
Verma et al. [34]	2011	India	Retrospective Study	26	Dengue IgM seropositivity	Partial recovery

Note: Abbreviations: IV: intravenous; IgM: immunoglobulin M; IgG: immunoglobulin G; NA: not available; CSF: cerebrospinal fluid; ELISA: enzyme-linked immunosorbent assay; RNA: ribonucleic acid electroencephalogram; NS1: nonstructural protein 1; WHO: World Health Organisation. Definitions. Full recovery: none or slight disability, Partial recovery: moderate or severe disability (may need help walking, numbness, tingling, may need ongoing assistance with daily activities).

**Table 2:** Characteristics of published studies included in the systematic review.

Study	Age	Sex	Number of TM Cases	Serotype	Treatment
Singh et al. [17]	45	Male	1	-	T9-11 laminectomy Evacuation of epidural haematoma. Multiple blood and platelet transfusions. Conservative management applied.
Ghosh et al. [18]	4		1	-	High dose methylprednisolone. Platelet transfusion and Packed red cell Supportive therapy for hepatitis and glomerulonephritis
Seet et al. [7]	44	Female	1	-	IV methylprednisolone 1 g for 5 days, Spinal MRI, Catheterisation for urinary retention, Intensive physiotherapy
Kunishige et al. [13]	42	Male	1	1	IV methylprednisolone Antibiotics
Fong CY et al. [19]	12	Female	1	-	IV methylprednisolone 30 mg/kg/day for 3 days followed by oral prednisolone IV Immunoglobulin (IVIG) 1 g/kg/day for 2 days. Intubated, 6 cycles of plasma exchange. Cervical epidural haematoma was managed conservatively
Gupta et al. [20]	26	Female	1	-	Methylprednisolone 1.0 mg/5 days Mechanical ventilation for 2 weeks
Wasay et al. [21]	18–35	5 females, 1 male	1	-	MRI/CT +/‒ EEG observations
Samanta et al. [22]		Male	1	primary/ secondary infection	Pulsed methylprednisolone Conservative therapy
Misra et al. [23]	5–70.	26 females, 90 males	1	1, 2 and 3	-
Sahu et al. [24]	25 +/‒ 18.3		7	-	Symptomatic treatment
Soars et al. [25]	11–79.	10 female, 3 male	2	1, 2 and 3	Corticosteroids IVIG
Weeratunga et al. [26]	Mean: 35	1 female, 6 male	2	-	Methylprednisolone pulsed 1 g/3 days
Puccioni-Sohler et al. (Brazil) [12]	22–74	6 females, 4 males	3	-	Methylprednisolone 1.0 mg/5 days. Additional Human IVIG 400 mg/kg/5 days for 1 patient
Larik et al. [10]	43	Male	1	-	IVIG
Lim et al. [27]	43	Male	1	-	IVIG
Tomar et al. [28]	42	Male	1	-	IV Methylprednisolone
Mo et al. [29]	65	Male	1	-	IV Methylprednisolone IVIG Plasma Exchange
Mota et al. [30]	21	Male	1	-	IV Methylprednisolone
Leão et al. [14]	58	Male	1	-	Ceftriaxone
Miranda de Sousa A et al. [31]	11	Female	1	-	IV Methylprednisolone 1g/day followed by prednisolone
Renganathan et al. [32]	14	Female	1	-	Symptomatic treatment
Chanthamat et al. [15]	61	Female	1	-	IV Methylprednisolone
Solomon et al. [33]			2	-	Symptomatic treatment
Sousa et al. [9]	Mean:34		26	3	IV methylprednisolone for 5 days
Verma et al. [34]		8 females, 18 males	1	-	IV Methylprednisolone

Note: Abbreviations: IV: intravenous; IVIG: intravenous immunoglobulin; TM: transverse myelitis; MRI: magnetic resonance imaging; CT: computed tomography; EEG: electroencephalogram; T9–11: thoracic vertebra 9 to 11; kg: kilogram.

## Appendix A

**Table A1:** FreeText and MeSH search terms.

Category	Search Terms
**Transverse Myelitis**	“transverse myelitis” or “myelitis” or “TM” or “*Myelitis, Transverse/”
**Dengue Virus**	“DEN” or “DHF” or “*Severe Dengue/” or “*Dengue Virus/*

**Table A2:** FreeText and MeSH search terms results.

Search	Results
**Transverse Myelitis Category**	146,970
**Dengue Virus Category**	83,794
**Transverse Myelitis AND Dengue Virus Category (ENG)**	226
**(Additional publications from contacts)**	16
**Duplicates Removal (62)**	180
**Limits Applied and Studies Removed (136)**	44
**Title and Abstract Exclusion (18)**	26
**Full Text Exclusion (1)**	25
